# Supplementary material for: Phytase dose-dependent response of kidney inositol phosphate levels in poultry
Source: PLoS One. 2022 Oct 19;17(10):e0275742. doi: 10.1371/journal.pone.0275742 (PMC9581429; doi:10.1371/journal.pone.0275742)
Supplement: Attachment — Submitted filename: Response to Reviewers.docx [file pone.0275742.s002.docx]

To whom it may concern,

Re. *Phytase dose-dependent response of kidney inositol phosphate levels in poultry*

*Authors: Colleen Sprigg^1^, Hayley Whitfield^1^, Emily Burton^2^, Dawn Scholey^2^, Michael R. Bedford^3^, Charles A. Brearley^1*^*

On behalf of all authors, I am pleased to return a revised manuscript that addresses the considered criticisms of our manuscript. We thank the reviewers and the editor for their inputs.

Our revisions address points raised by the Academic Editor:

1. We have edited our manuscript to meet PLOS ONE’s style requirements

2. Method of sacrifice, anaesthesia and efforts to alleviate suffering statement are provided in the text, new lines 128-149.

We additionally note:

The study was conducted feeding test materials previously investigated in a number of broiler studies and shown to cause no pain, suffering or lasting harm. The birds were not exposed to invasive experimental procedures during the study and received routine care and husbandry akin to a commercial broiler farm. No analgesia / anaesthetic was required, and no suffering occurred prior to death. The study was overseen by the site Named Animal Care and Welfare Officer, who is empowered to end the trial immediately if they believe any suffering occurs. Birds were health checked twice daily by a technical team not involved in the outcome of the study, and any sick or lame birds were euthanised immediately. A record table was used throughout the study to assure no deaths or illness were associated with any treatment and that mortality did not rise above the expected level for the age and strain of bird.

3. We have provided the raw data as excel files (Supporting Information)

To address the points raised by Reviewer #1:

L23 and L93-94: we have modified the description of the experimental design to emphasize the comparisons for which data is provided in this manuscript (new lines 22-23 and 92-95). Our reference to the use of titanium is limited to new lines 128-130.

L100: Table 1 has been corrected to include statement of calculated value, rather than measured; Crude fat and a clarifying footnote ^3^ states that Available phosphate does not account for the contribution of phytate P.

L107: the crude protein content is clarified as footnote ^1^

L114-115: is addressed in response to L93-94 above

L117: reference to inositol dose has been removed from the manuscript, because we restrict our analysis of effect of phytase dose. But, for our reviewer: in a typical diet there are approximately 2-2.5g of inositol per kg feed in the form of IP_6_ so if this were totally dephosphorylated through superdosing of phytase  it would yield approximately 2-2.5g inositol

L118: corrected

L124: again (see L117, reference to treatments not discussed in this manuscript (inositol and titanium) have been removed. This accommodates the concerns of Reviewers #2 and #3.

L142: we provide below (not in the text) a statement of our power calculations.

Data for mean gizzard and ileal inositol contents in responses to phytase additional with N and standard error reported by Walk et al., 2018 was used to conduct a power calculation indicating 6 replicates per treatment were sufficient to identify treatment differences at a power setting of 80% and a type 1 error rate of 5%.

[https://www.sciencedirect.com/science/article/pii/S0032579119309800](https://eur01.safelinks.protection.outlook.com/?url=https%3A%2F%2Fwww.sciencedirect.com%2Fscience%2Farticle%2Fpii%2FS0032579119309800&data=05%7C01%7CC.Sprigg%40uea.ac.uk%7C711ea01b6f8b4094d6fd08da2e81ac9f%7Cc65f8795ba3d43518a070865e5d8f090%7C0%7C0%7C637873432913400827%7CUnknown%7CTWFpbGZsb3d8eyJWIjoiMC4wLjAwMDAiLCJQIjoiV2luMzIiLCJBTiI6Ik1haWwiLCJXVCI6Mn0%3D%7C3000%7C%7C%7C&sdata=qJkLy9%2Fo3e4hOX6r64mXfpqP9N4lg8fZP7au5fHKMUg%3D&reserved=0)

Walk, C.L., Bedford, M.R. and Olukosi, OA. (2018) Effect of phytase on growth performance, phytate degradation and gene expression of *myo*-inositol transporters in the small intestine, liver and kidney of 21 day old broilers. Poultry Science 97(4) 1155-1162

L162-163: Tissue was sub-sampled from frozen organs, rather than extraction of the entire organ (new line 171). This approach was taken to allow trial development of methods appropriate for other classes of analyte not described in this study.

L166: corrected.

 L275: while researchers have noted empirical difference in eg. Ileal digestibility with different digestibility markers, it has not been appreciated by the animal nutrition field that that cell biology/signalling community have adopted the use of titanium dioxide as a solid phase extraction media for inositol phosphates. Indeed, we had cited the relevant methods (here in new lines 178-184 [32]). We will return to the discussion of the suitability of TiO2 as a digestibility marker, for inositol phosphate analysis in digesta and tissues in a subsequent manuscript. But, to simplify this manuscript, for the reader, in line with the preferences of Reviewers #2 and #3, we have limited our discussion in the revised manuscript to 3 treatments only (control, 500 and 6000FTU/kg ). We hope this ‘compromise’ meets the needs of all our reviewers.

To address the points raised by Reviewer #2:

1) and 2) we provide a more detailed explanation of inositol hexakisphosphate synthesis and inositol hexakisphosphate degradation by phytases (new lines 378-389) that argues at which point in metabolic sequences the diagnostic 2-phosphate is added or removed. The alternative, that inositol phosphates are taken up intact from the gut, cannot be discounted (and we say as much, new lines 382-383) but such a premise would necessarily be an invention since gut inositol phosphate transporters have not been described. Philosophically, the simplest explanation is the best – until transporters are identified.

2) We have removed all reference to treatments of the experimental design, for which data is not analysed.

We have corrected the assorted typos.

Line 25: we have corrected the sentence (new lines 25-26)

Lines 28-33: now addressed (see response to 1 and 2, above (new lines 378-389). We hope we have emphasized how isomeric identity (the 2-phosphate) is key to a full-understanding of the origins of isomers in tissues. A seminal reference [42] is cited (new line 378) to support the argument.

Line 109: corrected to Magnesium (new line 108)

Lines 114-122: we have amended the text to discuss the three treatments only (new lines 115-121).

Line 124: the titanium-containing diets have been removed from the analysis as requested (2) and Lines 114-122). We provide values of different IPs for the three diets in Table 2.

Lines 140 and 143, corrected

Lines 158-162: we have provided new details (new lines 153-169).

Lines 190-193: All details of our use of TiO2 beads to pre-concentrate and elute inositol phosphate (after Wilson et al [32]) are provided (new lines 178-192). In short, inositol phosphates bound to TiO2 under acid conditions can be eluted by change to alkaline pH.

Lines 203-207: the details of our statistical analyses (limited to Control, Phy500 and Phy6000 treatments) are provided (new lines 85-86 and 210-214).

Line 228: we, and others, have observed that soluble IPs transit through the GI tract faster than the insoluble marker and comment on this (new lines 275-279). We note that inositol transporters have been most widely studied in the small intestine of poultry and that inositol phosphate transporters have not been described (see also 1) and 2), above).

P values have been added to the table.

Lines 236-238: now corrected

Lines 271-277: text removed

Line 280: the Table has been corrected

Line 298: typo corrected.

Lines 295-296: corrected

Lines 298-300: full details of the comparisons made are provided (as probabilities) in Table 3

Line 338: corrected

Lines 394-425: we agree with the sentiment but the literature is sparse. Consequently, we leave our discussion of the topic to (new lines 455-561). We remain convinced that our data and this manuscript will encourage cell biologists to look at the poultry nutrition literature and the animal nutritionists to look at the cell biology of inositol phosphates.

A short paragraph: we hope the concluding sentences (new lines 455-461) make the point.

To address the points raised by Reviewer #3:

1.Line 93: we have now limited the statistical analysis to the three treatments Control, Phy500 and Phy6000 and removed confusing reference to other aspects of the feeding trial that are not reported here. This satisifies Reviewer #2 (Lines 114-122, 124). We retain mention of digestibility marker but emphasize it is not discussed further.

2. Lines 94-5: as above

3. Lines 114-122: now described (new lines 128-130 and made evident in Table 2)

4.: Table 2 is provided, we make no claim of significant difference

5.: Tables 3,4,5,6, now include summed inositol phosphates, all reported in the same unit

6.: Subtitles have been removed, the discussion remains largely the same.

7.: Figure 1 has been moved

8.: Table 4 and Table 5 and the associated description (new lines 266-279) makes the point that as phytase dose increases and inositol phosphates are reduced in the gizzard and ileal digesta so the gizzard and ileal inositol increases. This is consistent with the literature (new lines 285 and 398, references [9,20,27] and [9,16,17,23-27], respectively.

We thank our reviewers for their helpful comments on our manuscript.

Yours faithfully

Charles Brearley
