# Supplementary material for: Phytase dose-dependent response of kidney inositol phosphate levels in poultry
Source: PLoS One. 2022 Oct 19;17(10):e0275742. doi: 10.1371/journal.pone.0275742 (PMC9581429; doi:10.1371/journal.pone.0275742)
Supplement: Attachment — Submitted filename: Response to Reviewers.docx [file pone.0275742.s003.docx]

PONE-D-22-04744R1

Phytase dose-dependent response of kidney inositol phosphate levels in poultry

PLOS ONE

To The Editor,

We thank our reviewers for their consideration of our manuscript and the opportunity afforded to improve the manuscript further. We hereby respond to the comments of Reviewer #4:

1. Abstract was not associated with the results, especially L27-29.

We think the reviewer has not appreciated that L27-29 make reference to the results described in lines 374-382: paragraph beginning, ‘ The peaks identified in Fig 1B…’ and further elaborated in the Discussion, paragraphs 1 and 2, lines 386-411.

There was no linear or quadratic analysis in the statistical analysis part. At the same time, no P value of liner relationship was shown in results table.

The reviewer is correct, testing makes clear that the relationship is non-linear, we have therefore removed references to linearity – changing the term to one ‘progressive’- that does not need statistical validation.

2. How many birds were used in this study? The number was not correct. The trial as a whole was a 2 x 4 factorial design, of 8 treatments, designed to test 3 separate hypotheses. Data presented in this study speaks to only 3 of these treatment groups, with 60 birds per treatment in 6 pens of 10 birds per pen, totalling 180 birds.

This has been further clarified in lines 146-149.

3. In introduction, the hypothesis of this study was not clear. From L67 to L80, these parts had no strong relationship with the object of the study.

We have clarified this by addition of a sentence, lines 81-83, beginning, ‘It was hypothesised that ….’.

4. L85 what kind of design? 2*4 or 3 treatments? So many statements of experiment design were not consistent.

This has been further clarified in lines 146 to 149

5. there was no direct results of plasma vitamin D content in this study. L475-L482 the discussion of vitamin D metabolism in chicken was not suitable with less logic.

We choose to retain the Discussion unaltered and make the point that the Discussion puts our results in context of calcium and phosphate homeostasis and inter-organ axis for its control. Put simply, we are the first to describe effect of phytase on the inositol phosphates of any tissue, let alone kidney. That the kidney is central to phosphate and calcium homeostasis deserves the discussion of how the kidney is involved in calcium and phosphate homeostasis. We do believe that the assorted readers of this manuscript will hold a range of perspectives that make our Discussion relevant to their interests.

6. L494-L503 which results was discussed in this paragraph? Many references mentioned mRNA expressions during calcium and phosphate metabolism

We think the reviewer has not appreciated that Discussion, paragraph 7 specifies a study (Greene et al. (15)) that describes effects of phytase on inositol phosphate synthesis genes. These genes should influence inositol phosphate levels and our study measures inositol phosphates in tissues.

what’s the relationship with the results? Discussion, paragraphs 2 and 3 explicitly places the observed results for kidney inositol phosphate changes in the context of phosphate homeostasis under the prevailing sufficient phosphate and calcium provision conditions of this study.

7. in conclusion part, this study suggested that TiO2 was a better preconcentration of method for inositol phosphate analysis, but less discussion was mentioned. Thus, the conclusion of this study need rewrite.

We have expanded the last sentence of the last paragraph of Discussion, but retain the focus on the results that this method reveals (rather than the method itself, which we properly report as having been published previously by Wilson et al. 2015, but which has not been used for tissue/organ purpose).

Finally, we reiterate the comments of Reviewer #5: I believe the authors have addressed all the comments adequately, and I have no further comments as a reviewer reviewed the respsonses of the authors to the other reviewers' comments.

Yours faithfully

Charles Brearley
